# *α*-(1,4)-Amylase, but not *α*- and *β*-(1,3)-glucanases, may be responsible for the impaired growth and morphogenesis of *Paracoccidioides brasiliensis* induced by *N*-glycosylation inhibition

**DOI:** 10.1002/yea.2983

**Published:** 2013-11-28

**Authors:** Fausto Bruno Dos Reis Almeida, Laurine Lacerda Pigosso, André Ricardo de Lima Damásio, Valdirene Neves Monteiro, Célia Maria de Almeida Soares, Roberto Nascimento Silva, Maria Cristina Roque-Barreira

**Affiliations:** 1 Departamento de Biologia Celular e Molecular e Bioagentes Patogênicos, Faculdade de Medicina de Ribeirão Preto, Universidade de São PauloAv. Bandeirantes 3900, Ribeirão Preto, SP, 14049-900, Brasil; 2 Laboratório de Biologia Molecular, Instituto de Ciências Biológicas, Universidade Federal de GoiásGoiânia, GO, Brasil; 3 Laboratório Nacional de Ciência e Tecnologia do Bioetanol, Centro Nacional de Pesquisa em Energia e MateriaisBrasil; 4 Universidade Estadual de Goiás, UnUCETBR 153, Km 98. Campus Henrique Santillo, Anapolis, GO, 75000-000, Brasil; 5 Departamento de Bioquímica e Imunologia, Faculdade de Medicina de Ribeirão Preto, Universidade de São PauloAv. Bandeirantes 3900, Ribeirão Preto, SP, 14040-900, Brasil

**Keywords:** *N*-glycan, *Paracoccidioides brasiliensis*, glucanase, amylase, cell wall

## Abstract

The cell wall of *Paracoccidioides brasiliensis*, which consists of a network of polysaccharides and glycoproteins, is essential for fungal pathogenesis. We have previously reported that *N*-glycosylation of proteins such as *N*-acetyl-*β*-d-glucosaminidase is required for the growth and morphogenesis of *P. brasiliensis*. In the present study, we investigated the influence of tunycamicin (TM)-mediated inhibition of *N*-linked glycosylation on *α*- and *β*-(1,3)-glucanases and on *α*-(1,4)-amylase in *P. brasiliensis* yeast and mycelium cells. The addition of 15 µg/ml TM to the fungal cultures did not interfere with either *α*- or *β*-(1,3)-glucanase production and secretion. Moreover, incubation with TM did not alter *α*- and *β*-(1,3)-glucanase activity in yeast and mycelium cell extracts. In contrast, *α*-(1,4)-amylase activity was significantly reduced in underglycosylated yeast and mycelium extracts after exposure to TM. In spite of its importance for fungal growth and morphogenesis, *N*-glycosylation was not required for glucanase activities. This is surprising because these activities are directed to wall components that are crucial for fungal morphogenesis. On the other hand, *N*-glycans were essential for *α*-(1,4)-amylase activity involved in the production of malto-oligosaccharides that act as primer molecules for the biosynthesis of *α*-(1,3)-glucan. Our results suggest that reduced fungal *α*-(1,4)-amylase activity affects cell wall composition and may account for the impaired growth of underglycosylated yeast and mycelium cells. © 2013 The Authors. Yeast published by John Wiley & Sons Ltd.

## Introduction

The dimorphic fungus *Paracoccidioides brasiliensis* is the causative agent of paracoccidioidomycosis, a systemic granulomatous disease that is the most prevalent systemic mycosis in Latin America. The disease is triggered by inhalation of airborne propagules from fungal mycelia, which convert to the yeast form inside the host. The yeast form is infectious and develops at 37 °C (Brumer *et al*., 1993). The lungs are the primary sites of infection, and yeasts disseminate from the lungs to other organs and cause systemic disease (Franco *et al*., 1994).

The cell wall of *P. brasiliensis*, similar to that of many other fungi, is a network of glycoproteins and polysaccharides that protects the fungal cell from environmental stress (De Groot *et al*., 2005) and confers virulence to the fungus. These constituents play important roles in fungal growth, because the polysaccharide network requires continuous remodelling. Glucans account for approximately 40% of the cell wall in the mycelium and yeast forms (Kanetsuna *et al*., 1969). *α*-Glucan is the major cell wall glucan of the yeast form, whereas the mycelial form contains larger amounts of *β*-glucan (Kanetsuna and Carbonell, 1970; San-Blas and San-Blas, 1977). Therefore, variations in cell wall glucans may play key roles in the dimorphism of the fungus and, thus, its pathogenesis.

*N*-glycans attached to asparagine residues (i.e. inserted into the well-known sequon Asn–Xaa–Ser/Thr, in which Xaa denotes any amino acid except proline) (Bause, 1983) are usually involved in protein folding, intracellular transport and protection from proteolytic degradation (Nagai *et al*., 1997). The *N*-glycans of *P. brasiliensis* proteins have recently been identified as essential for the growth and morphogenesis of the fungi and for some biological activities of yeast proteins (Dos Reis Almeida *et al*., 2011). *N*-glycosylation can be altered by a number of products, including tunicamycin (TM). TM is a nucleoside antibiotic that inhibits *N*-glycosylation by blocking the transfer of uridine diphosphate-*N*-acetyl-glucosamine to dolicholphosphate – the first step in the synthesis of the dolichol-linked oligosaccharide – thereby decreasing the formation of dolichol-pyrophosphoryl-*N*-acetylglucosamine (Elbein, 1987; Varki *et al*., 1999). We have verified that TM added to cultures at a final concentration of 15 µg/ml inhibits *N*-glycosylation of *P. brasiliensis* yeasts proteins, because the glycoprotein electrophoretical profiles of the extract of yeasts grown in these cultures were similar to those of yeast extracts digested with peptide *N*-glycosidase F (PNGase-F) (Dos Reis Almeida *et al*., 2011). The results of the present study validate the use of TM to evaluate the effect of *N*-glycosylation inhibition on the activity of *N*-linked glycoproteins such as fungal glycosyl hydrolases, which are components of the *P. brasiliensis* yeast extract.

Among *P. brasiliensis* glycosyl hydrolases, *α*-(1,3)-glucanase (EC 3.2.1.59) hydrolyses the *α*-glucan chain, a polymer with a branched structure predominantly containing *α*-linked glucose residues that is imperative for yeast virulence (San-Blas *et al*., 1977). The literature contains few reports on the characterization of fungal *α*-(1,3)-glucanases (Dekker *et al*., 2004; Marion *et al*., 2006) and no reports on enzyme glycosylation. Likewise, *β*-(1,3)-glucanase (EC 3.2.1.39) hydrolyses the *β*-glucan chain, a polymer consisting of *β*-linked glucose residues, which is largely predominant in mycelial forms of *P. brasiliensis*. *β*-(1,3)-Glucanase plays key roles in morphogenetic-morpholytic processes during fungal development and differentiation (Adams, 2004). *α*-(1,4)-Amylases (EC 3.2.1.1) are glycosyl hydrolases that randomly cleave the *α*-glucosidic bonds in starch. This reaction produces linear and branched oligosaccharides of various sizes (Van der Maarel *et al*., 2002). Some studies have associated *α*-(1,4)-amylases with cell wall *α*-(1,3)-glucan generation and modification (Davies and Wilson, 1999), as recently shown in *Histoplasma capsulatum* (Marion *et al*., 2006) and *P. brasiliensis* (Camacho *et al*., 2012).

We have previously reported the importance of *N*-glycans for the growth and morphogenesis of *P. brasiliensis* yeasts as well as for enzymatic activities implicated in chitin metabolism (Dos Reis Almeida *et al*., 2011). Therefore, we decided to examine whether *N*-glycosylation could contribute to the biochemical properties of *α*-(1,3)-glucanase, *β*-(1,3)-glucanase and *α*-(1,4)-amylase in *P. brasiliensis* yeast and mycelium cells. We found that *N*-glycans may not be required for the production or secretion of *α*- or *β*-(1,3)-glucanases in *P. brasiliensis* yeast cells and that they do not interfere with the function of these enzymes; however, *N*-glycans are important for *α*-(1,4)-amylase activity, which is involved in the biosynthesis of fungal cell wall *α*-(1,3)-glucan (Camacho *et al*., 2012). Furthermore, this study confirmed that *α*-(1,3)-glucanase is found in *P. brasiliensis* yeast cells, whereas *β*-(1,3)-glucanase is found in mycelium cells.

## Materials and methods

### Strain and growth conditions

*P. brasiliensis* isolate Pb18 was used in all experiments. The yeast and mycelial phases were maintained *in vitro* by subculturing the cells every 7 days at 36 °C and 22 °C, respectively, in Fava Netto’s semi-solid medium containing (w/v) 1% peptone, 0.5% yeast extract, 0.3% proteose peptone, 0.5% beef extract, 0.5% NaCl, 4% glucose and 1.2% agar, at pH 7.2. To ensure the maintenance of Pb18 virulence, we performed serial passages in BALB/c mice before isolates were used in experiments.

TM (Sigma, St. Louis, MO, USA) stock solution (10 mg/ml) was prepared in 20 mm NaOH. *P. brasiliensis* was harvested after 48 h, and the cells were washed twice with 10 mm phosphate buffer and incubated in liquid YPD medium (1% yeast extract, 2% peptone and 2% dextrose, all w/v) with 15 µg/ml TM for 3 days. In control preparations, 20 mm NaOH was added to the medium instead of TM.

For the growth of yeast and mycelia and the mycelia-to-yeast and yeast-to-mycelia transitions, cultures were maintained in a liquid YPD medium at suitable temperatures (i.e. 36 °C for yeast and 25 °C for mycelium) on a rotary shaker at 100 rpm for 72 h. Yeast cells were harvested via centrifugation at 2000 × *g* for 10 min at 4 °C; the supernatant was designated the extracellular crude extract. Subsequently, the pellet was washed twice with phosphate-buffered saline (PBS) containing a protease inhibitor cocktail for fungal and yeast cells (Sigma, cat. no. P 8215). Disruption was performed via sonication with three pulses of 60 s each, as previously described (Dos Reis Almeida *et al*., 2010). The tested samples (fully glycosylated and underglycosylated) were corrected to the same final protein concentration, as previously described (Dos Reis Almeida *et al*., 2011).

For the transition experiment (mycelium-to-yeast or yeast-to-mycelium), we observed (on a smaller scale) that TM did not block differentiation, thus demonstrating the viability of the study. After 72 h of growth, *P. brasiliensis* yeast or mycelia cells were harvested, washed twice with 10 mm phosphate buffer and incubated in liquid YPD medium containing 15 µg/ml TM. The cells were then incubated for 72 h at the corresponding temperature for transition. In control preparations, 20 mm NaOH was added to the medium. The cells were collected via centrifugation at 10 000 × *g* for 15 min at 4 °C, and extraction buffer (20 mm Tris–HCl, pH 8.8, and 2 mm CaCl_2_) containing a mixture of nuclease and protease inhibitors (Sigma) was added to the material. This suspension was distributed in extraction tubes with glass beads and processed in a bead beater apparatus (BioSpec Products, Bartlesville, OK) for five cycles of 30 s on ice. The supernatant was collected and concentrated in an Amicon UltraCentrifugal Filter (10 kDa; Millipore, Bedford, MA, USA) and designated the intracellular crude extract. The supernatant was filtered through 0.22 µm filters, added to a mixture of nuclease and protease inhibitors and concentrated in the Amicon UltraCentrifugal Filter.

### Cell viability

The viability of fungal suspensions was determined through fluorescein diacetate/ethidium bromide staining, as described previously (Calich *et al*., 1979). Only cultures with viability > 85% were used.

### Preparation of glucans

*α*-Glucan was obtained as previously described (Takehara *et al*., 1981), with slight modifications. *Aspergillus niger* was obtained from the enzymology group collection at the Federal University of Goiás and grown at 30 °C for 48 h in MYG medium containing (w/v) 0.5% malt extract, 0.25% yeast extract and 1% glucose. Cells were harvested via continuous centrifugation (5000 × *g*, 5 min) and solubilized in 3 m KOH via heating at 100 °C for 2 h. The resulting solution was centrifuged at 13 000 × *g* for 30 min to remove insoluble materials. The insoluble pellets were again subjected to KOH treatment. The supernatants obtained through the alkali treatment were combined, neutralized with glacial acetic acid and mixed with an equal volume of methanol to precipitate solubilized glucans. After five washings with a 50% v/v methanol solution, the precipitates were suspended in distilled water and freeze-dried. The cell-associated, alkali-soluble glucans of *A. niger* were suspended in a 0.02% w/v sodium azide solution and stored at 4 °C until use. Carbohydrate content (*α*- and *β*-[1,3]-glucan) was verified using a phenol–sulphuric acid method, as previously described (Dubois *et al*., 1951, 1956), and adapted to the method-in-microplate assay (Masuko *et al*., 2005).

### *β*-(1,3)-Glucanase assay

*β*-(1,3)-Glucanase assay was performed as described previously (Ramada *et al*., 2010), using laminarin (Sigma) as the substrate. The amount of reducing sugar released from laminarin was determined as previously described (Miller, 1959).

### *α*-(1,3)-Glucanase assay

*α*-(1,3)-Glucanase assay was performed as previously described (Takehara *et al*., 1981), with slight modifications, using the *α*-glucan from *A. niger* as the substrate. The assay was performed by mixing 10 µl enzyme solution, 10 µl PBS buffer, pH 5.5, and 20 µl 0.2% w/v *α*-glucan in a polymerase chain reaction microplate. The mixture was incubated at 37 °C for 10 min. After incubation, 100 µl 3,5-dinitrosalicylic acid reagent was added and the mixture was heated to 95 °C for 5 min, then cooled to 25 °C for 2 min. Subsequently, 100 µl of the reaction mixture was transferred to an enzyme-linked immunosorbent assay microplate. The absorbance was measured at 540 nm using a microplate reader. One unit of enzyme activity was defined as the amount of enzyme required to produce 1 µm reducing sugars/min. The enzyme activity values are the mean values of at least three replicates. The protein concentration was measured using the bicinchoninic acid assay (Pierce Chemical Co., Rockford, IL, USA), with bovine serum albumin (BSA) as the standard.

### *α*-(1,4)-Amylase assay

*α*-(1,4)-Amylase activity was determined by monitoring starch hydrolysis according to the method of Fuwa (1954). One unit of *α*-(1,4)-amylase activity was defined as the amount of enzyme required to hydrolyse 0.1 mg starch/min.

### Enzymatic characterization

The pH effect on *α*- and *β*-(1,3)-glucanase activities was determined by varying the pH of the reaction mixtures using 0.1 m phosphate citrate buffer, pH 2.5–7.0, or 0.1 m sodium phosphate buffer, pH 7.5–8.5. The effect of temperature on *α*- and *β*-(1,3)-glucanase activities at optimum pH was determined by varying the temperature of the reaction in the range 20–65 °C.

*α*-(1,4)-Amylase was characterized as previously described (De Barros *et al*., 2009). The optimum pH was determined by varying the pH of the reaction mixtures using the following buffers (100 mm): sodium acetate, pH 2.5–5.5; sodium phosphate, pH 6.0–7.0; and Tris–HCl, pH 7.5–8.0. The optimal temperature for *α*-(1,4)-amylase was determined by varying the temperature (20–70 °C) in a 100 mm sodium acetate buffer, pH 5.5.

The Michaelis–Menten constant (*K*_m_) was determined using GraphPad Prism software v. 5.00. Non-linear regression analysis of the data was obtained by measuring the rate of *α*- and *β*-(1,3)-glucan hydrolysis, using a range of 0.5–5.0 mm for *α*- and *β*-(1,3)-glucanases, and by measuring the rate of starch hydrolysis, using a range of 0–1.0 15 mg/ml for *α*-(1,4)-amylase.

### Optical microscopy

For optical microscopy studies, the samples were observed with a Medilux optical microscope at × 40 magnification.

### Prediction of three-dimensional (3D) structures and *N*-glycosylation sites

3D structures were predicted using the I-TASSER server (Roy *et al*., 2010). The Protein Data Bank (PDB) files were uploaded to GLYCAM WEB (http://www.glycam.ccrc.uga.edu) to attach the *N*-glycans at previously predicted sites. Man5GlcNAc2 was the pattern oligosaccharide attached to the structures (Weerapana and Imperiali, 2006).

### Statistical analysis

Data are either the means of, or representative results from, at least three independent experiments, each performed in triplicate. Comparisons and statistical analysis were performed using Graphpad Prism Software v. 5.00. The Turkey’s multiple and one-way ANOVA comparison post-test were applied. *p* < 0.05 was considered statistically significant; *p < 0.05, **p < 0.01 and ***p < 0.001.

## Results

**Figure 1:**
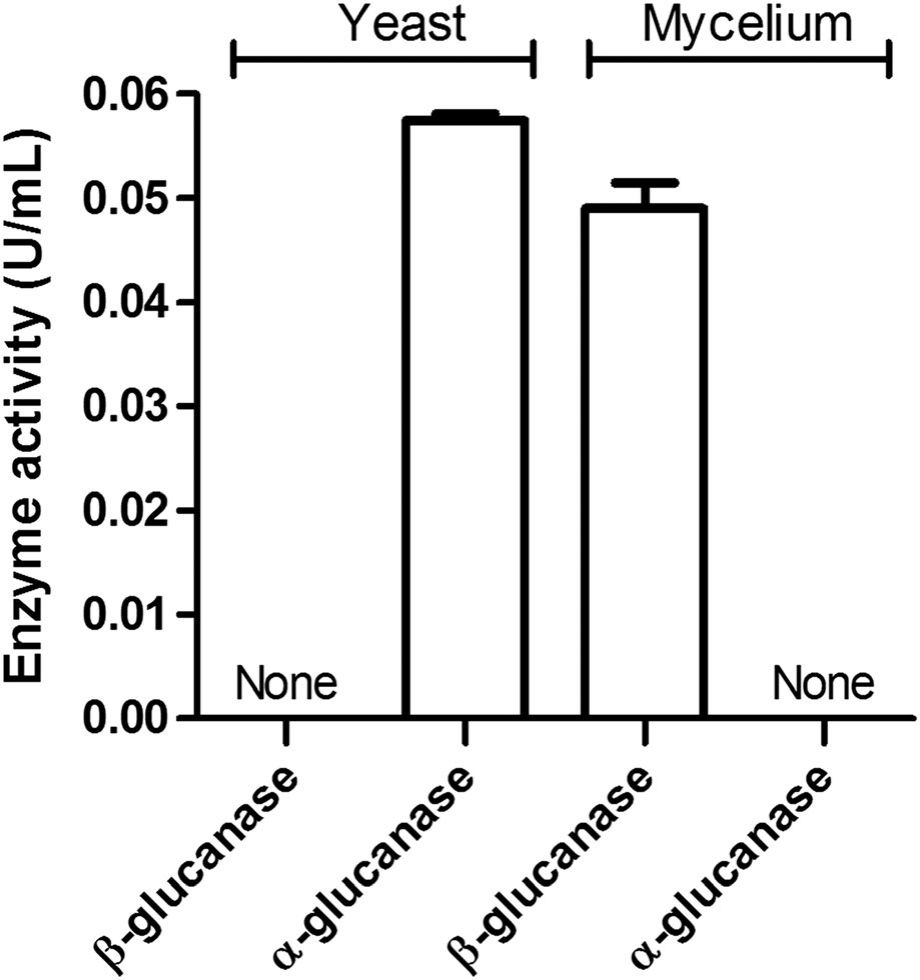
Detection of *α*- and *β*-(1,3)-glucanase activities in *Paracoccidioides brasiliensis* yeast and mycelium cells. *Paracoccidioides brasiliensis* yeast cells and mycelia were disrupted and the extracts obtained were assayed for *α*- and *β*-(1,3)-glucanase activities through detection of *α*- and *β*-(1,3)-glucan hydrolysis, using the appropriate substrate and 3,5-dinitrosalicylic acid reagent. One unit of enzyme activity was defined as the amount of protein required to produce 1 µm glucan in 1 min. The enzyme activity values correspond to the mean values of at least three replicates

We first validated our test for *α*- and *β*-(1,3)-glucanase detection by assaying the activities of these enzymes in crude extracts from whole mycelia or yeast cells of *P. brasiliensis*. In the yeast cell extract, we detected a significant level of *α*-(1,3)-glucanase activity, whereas *β*-(1,3)-glucanase activity was not detected (Figure 1). By contrast, only *β*-(1,3)-glucanase activity was significantly detected in the extract of *P. brasiliensis* mycelium cells (see Figure 1). These results agree with reports that in yeasts 95% of the glucans are *α*-linked, whereas in mycelia all glucans are *β*-linked (reviewed by Puccia *et al*., 2011).

**Figure 2:**
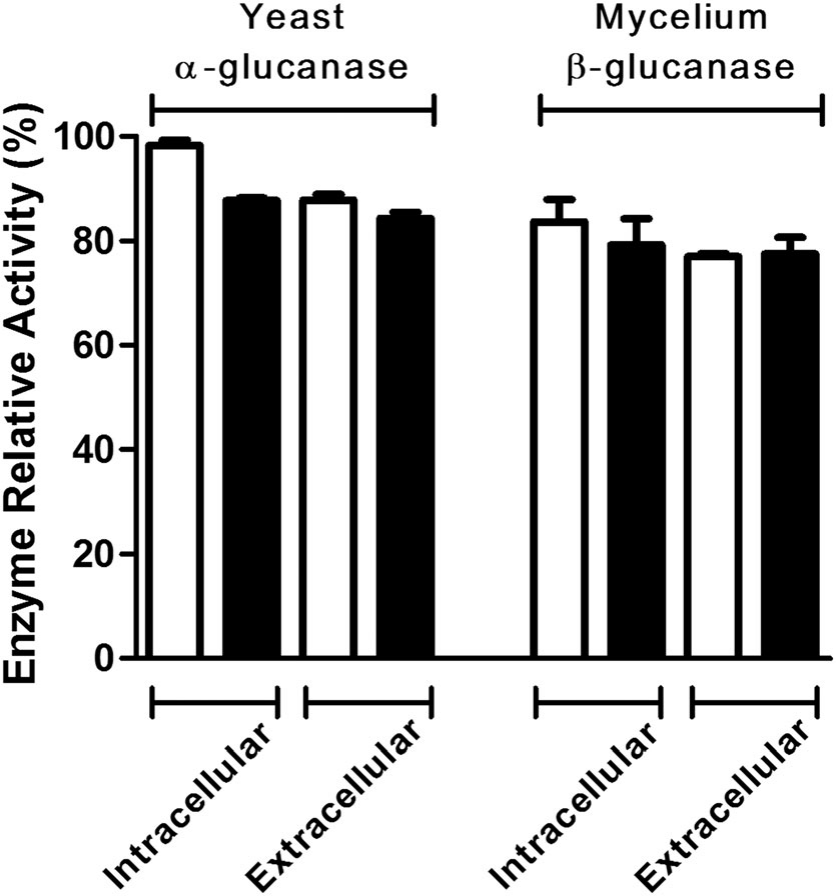
Effect of tunicamycin on *α*- and *β*-glucanase activities and their secretion in *P. brasiliensis* yeast and mycelium cells. *P. brasiliensis* cells, cultured in the absence (white bars) or presence (black bars) of 15 µg/ml tunicamycin, were centrifuged to obtain the fungal proteins secreted by yeast and mycelium cells (extracellular). The disrupted yeast and mycelium cells provided the intracellular extracts. *α*- or *β*-(1,3)-Glucan hydrolysis was detected using the appropriate substrate and 3,5-dinitrosalicylic acid reagent. Maximum activity was standardized as 100%, and the enzyme activity corresponds to the mean values of at least three replicates

To verify the importance of *N*-glycan on *α*- and *β*-(1,3)-glucanase activities in *P. brasiliensis*, we compared their production by mycelia and yeast cells cultivated in the presence or absence of 15 µg/ml TM. This concentration was chosen based on a previous report that it strongly inhibits the *N*-glycosylation process (Dos Reis Almeida *et al*., 2011). Similar levels of enzymes activity were detected in extracts of disrupted cells (intracellular) from TM-treated (underglycosylated) or untreated (fully glycosylated) *P. brasiliensis* yeast or mycelium. We also observed that the levels of *α*- and *β*-(1,3)-glucanase activities detected in the yeast and mycelia culture supernatants (extracellular) were similar to the respective intracellular levels and were unaffected by TM treatment (Figure 2). These data indicate that *N*-glycans may not be required for *α*- and *β*-(1,3)-glucanase activities or secretion.

**Figure 3:**
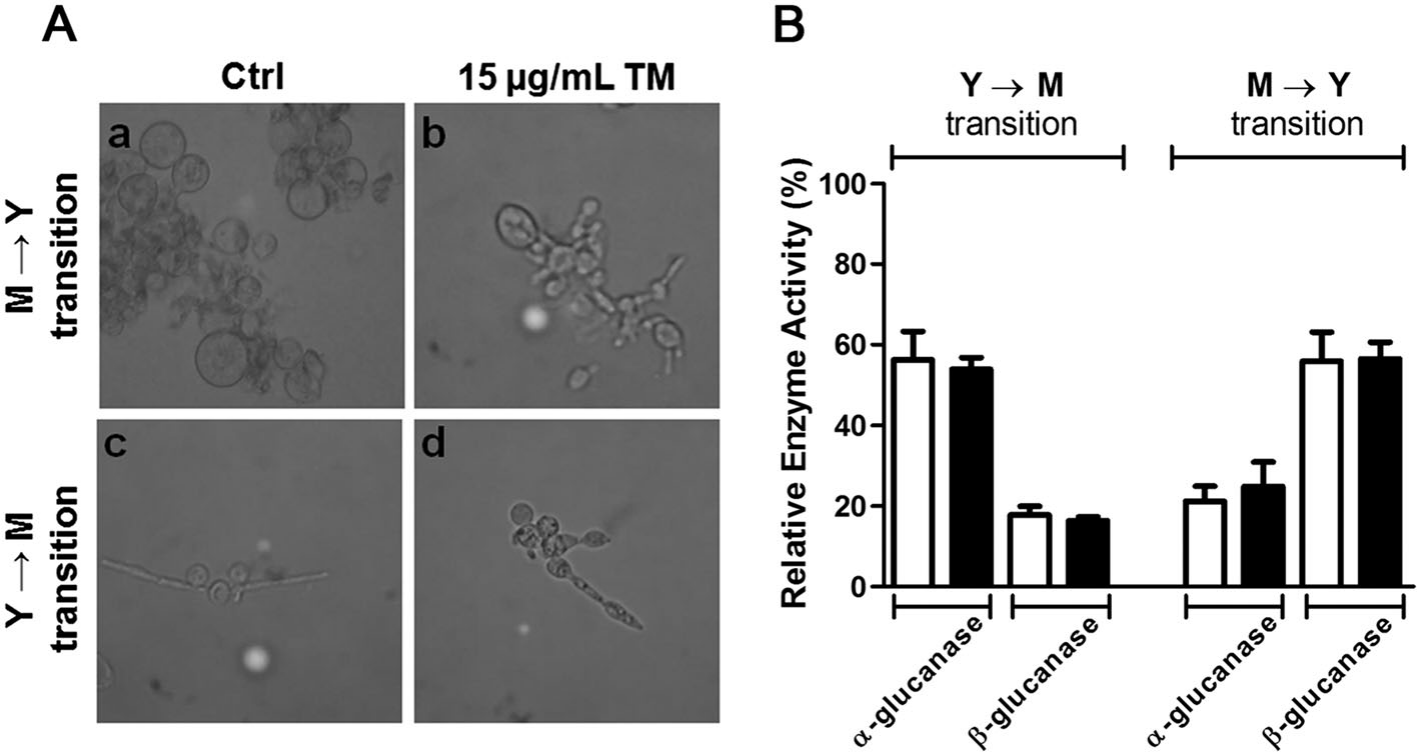
Effect of tunicamycin (TM) on the transition of *P. brasiliensis* cells and the glucanases activities in the extracts obtained from the transition forms. *Paracoccidioides brasiliensis* mycelia and yeasts were grown in the absence (Ctrl) or presence of 15 µg/ml TM for 72 h at 37 °C (a, b) and 25 °C (c, d) to obtain fungal transition forms. The fungal forms were examined by optical microscopy (A) and their extracts were assayed for *α*- and *β*-(1,3)-glucanases (B). The morphology of fungal forms obtained from the transition of mycelium to yeast (M → Y) and yeast to mycelium (Y → M) are shown in panels a/b and c/d, respectively. The *α*- or *β*-(1,3)-glucanase relative activities were detected using the appropriate substrate and 3,5-dinitrosalicylic acid reagent and expressed as a percentage of the maximum activity (100%), which was the *α*-(1,3)-glucanase activity exerted by the *P. brasiliensis* yeast extract. The enzymes activity corresponds to the mean value of at least three replicates

To investigate the effects of *N*-glycosylation inhibition on fungal transition, we treated *P. brasiliensis* yeast and mycelium with 15 µg/ml TM and cultivated them for 72 h at 25 °C and 37 °C, respectively. They were examined for microscopic features (Figure 3A) and *α*- and *β*-(1,3)-glucanase activities, which were measured in the extract obtained from the disrupted fungal forms (Figure 3B). In the absence of TM, the transition of mycelium to yeast (M → Y) showed spherical structures identified as chlamidospore-like cells that produced few buds (Figure 3Aa), whereas in the presence of TM, pseudohyphae were the largely prominent form (Figure 3Ab). The transition of yeast to mycelium (Y → M) showed mycelial-like morphotypes with rare fungal spherical forms (Figure 3Ac). By contrast, the forms cultured in the presence of TM appeared as chains of elongated yeast-like cells (Figure 3Ad). These results indicate that TM treatment interfered in transition in both directions, mycelium-to-yeast and yeast-to-mycelium. Indeed, a delayed transition was verified by accompanying the cultures in terms of colony-forming unit (CFU) numbers, optical density readings and morphology of the growing forms (data not shown). The observations we made are consistent with our previous report on the effect of TM on yeast growth and morphogenesis of *P. brasiliensis* (Dos Reis Almeida *et al*., 2011). The detection of *α*- or *β*-(1,3)-glucanase activities in these transitions showed that both activities coexisted in the two transition forms. Comparison of the relative activity levels of these enzymes showed that *α*-(1,3)-glucanase activity was three times higher in yeast-to-mycelium transition forms, whereas *β*-(1,3)-glucanase was three times higher in mycelium-to-yeast transition forms (see Figure 3B).

To evaluate the influence of inhibition of protein *N*-glycosylation on fungal enzyme features, we assayed extracts from TM-treated (underglycosylated) or untreated (fully glycosylated) *P. brasiliensis* yeast and mycelium under similar protein concentrations for *α*- and *β*-(1,3)-glucanase activities. Both fully glycosylated and underglycosylated preparations at pH 5.5 and 45 °C showed similar levels of *α*- and *β*-(1,3)-glucanase activities (data not shown). In addition, the kinetic parameters of these activities were determined for both preparations. Solutions of *α*-(1,3)-glucan, and *β*-(1,3)-glucan, which had similar carbohydrate concentrations as determined by the phenol–sulphuric acid method (Dubois *et al*., 1951, 1956; Masuko *et al*., 2005), were used as substrates for *α*- and *β*-(1,3)-glucanases contained in both fully and underglycosylated extracts of *P. brasiliensis* yeasts. Apparent *K*_m_ and *V*_max_ were determined from non-linear regression analysis of data obtained by measuring the rate of *α*- and *β*-(1,3)-glucan hydrolysis (0.5–5.0 mm). The *α*-(1,3)-glucanase *V*_max_ of the fully glycosylated preparation (0.72 U/mg) was similar to that of the underglycosylated extract (0.71 U/mg). For *β*-(1,3)-glucanase also, the fully glycosylated and underglycosylated preparations showed similar *V*_max_ values (0.58 and 0.54 U/mg, respectively; Table 1). In addition, the *K*_m_ of the *α*-(1,3)-glucanase in the fully glycosylated extract was slightly lower (1.90 mm) than that in the underglycosylated preparation (2.26 mm). The *K*_m_ of the *β*-(1,3)-glucanase in the fully glycosylated extract was also slightly lower (1.65 mm) than that in the underglycosylated preparation (1.87 mm). These findings indicate that the *N*-linked glycosylation of *α*- and *β*-(1,3)-glucanases does not influence their catalytic activity.

**Table 1:** Partial kinetic characterization of *α*-(1,3)-glucanase, *β*-(1,3)-glucanase, and *α*-(1,4)-amylase activities contained in the extract of *Paracoccidioides brasiliensis* yeast and mycelial cells that were cultured in the absence (fully glycosylated extract) or presence (underglycosylated extract) of tunicamycin

	*K*_mapp_ (mm)	*V*_maxapp_
*α*-(1,3)-Glucanase		
Fully glycosylated	1.90 ± 0.62	0.72 ± 0.10
Underglycosylated	2.26 ± 0.90	0.71 ± 0.13
*β*-(1,3)-Glucanase		
Fully glycosylated	1.65 ± 0.54	0.58 ± 0.07
Underglycosylated	1.87 ± 0.73	0.54 ± 0.09
*α*-(1,4)-Amylase		
Fully glycosylated	0.46 ± 0.16	1.02 ± 0.12
Underglycosylated	0.80 ± 0.18	0.79 ± 0.07

Data represent three independent assays.

*p* < 0.05 just between fully glycosylated and underglycosylated *α*-(1,4)-amylase.

**Figure 4:**
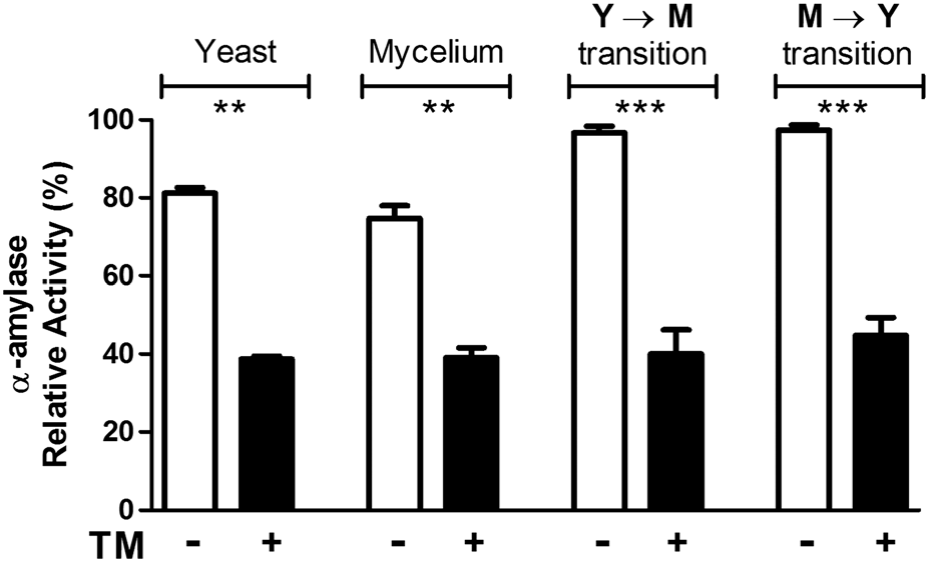
Effect of tunicamycin on the *α*-(1,4)-amylase activity in the extracts of yeast, mycelium and transition forms of *P. brasiliensis*. *P. brasiliensis* mycelia and yeasts were grown in the absence (Ctrl, white bars) or presence (black bars) of 15 µg/ml tunicamycin for 72 h at 37 °C and 25 °C to obtain the fungal transition forms (M → Y) and (Y → M), respectively. The transition forms were disrupted and assayed for to *α*-(1,4)-amylase activity, which was detected by using the appropriate substrate and 3,5-dinitrosalicylic acid reagent. The results were expressed as percentage of the maximum enzymatic activity, standardized as 100%. Data represent three independent assays. **p < 0.01 and ***p < 0.001

Finally, we evaluated the influence of *N*-glycosylation inhibition promoted by TM treatment on fungal *α*-(1,4)-amylase activity, which was measured in extracts with protein concentrations similar to those obtained through disruption of yeasts, mycelium or transition forms of yeast-to-mycelium or mycelium-to-yeast after culture in the absence or presence of TM. Samples from TM-treated *P. brasiliensis* cells displayed *α*- (1,4)-amylase activity at least two-fold lower than that in control cells (Figure 4). Fully and underglycosylated preparations exhibited maximum *α*-(1,4)-amylase activity at pH 5.5 and 50 °C (data not shown). Under these optimal conditions, the fully glycosylated preparation exhibited *α*-(1,4)-amylase activity approximately three times higher than that exhibited by the underglycosylated preparation. The apparent *K*_m_ of the *α*-(1,4)-amylase in the fully glycosylated preparation (0.46 mm) was substantially lower (*p <* 0.05) than that of *α*-(1,4)-amylase in the underglycosylated extract (0.80 mm). Finally, the apparent *V*_max_ of the *α*-(1,4)-amylase contained in the fully glycosylated extract (1.02 U/mg) was higher (*p <* 0.05) than that contained in the underglycosylated preparation (0.79 mm).

**Figure 5:**
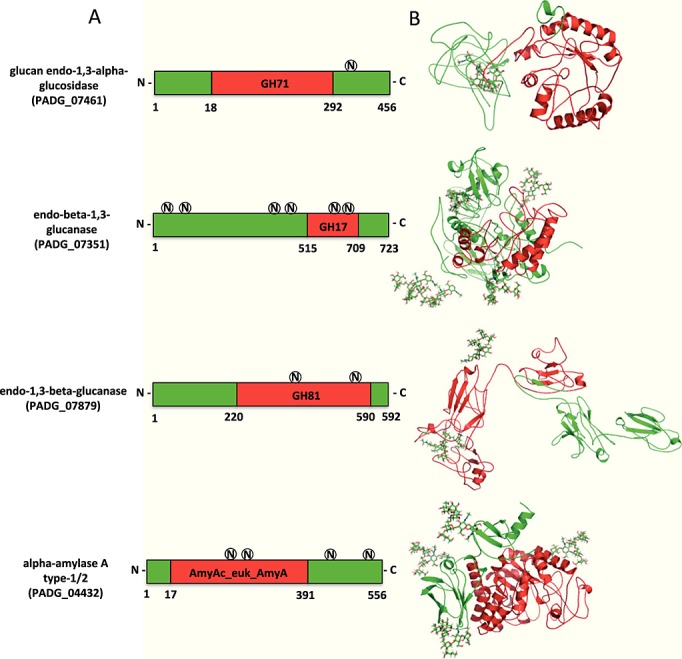
Predicted 3D structures of *P. brasiliensis α*-(1,3)-glucanase, *β*-(1,3)-glucanase and *α*-(1,4)-amylase. (A) Proteins representing the assigned domains for the glycosyl hydrolase (GH) family and *N*-glycosylation positions. The circled letters (N) represent the predicted *N*-glycosylation sites. (B) Glycoprotein 3D structures predicted by I-TASSER, NetNGlyc and GLYCAM WEB (see text). The GH domains are highlighted in red

Using the genome database from the Broad Institute (http://www.broadinstitute.org/annotation/genome/paracoccidioides_brasiliensis/MultiHome.html), we found the gene *PADG_07461*, predicted to encode for *α*-(1,3)-glucanase, and the genes *PADG_07351* and *PADG_07879*, predicted to encode for *β*-(1,3)-glucanases, in *P. brasiliensis* Pb18 (Figure 5A). *PADG_07461*, with an open reading frame sequence of 1371 nucleotides, translates into a sequence of 457 amino acids with an isoelectric point (pI) of 8.15 and a molecular weight (MW) of 51 kDa. As predicted using the NetNGlyc 1.0 Server tool (Gasteiger *et al*., 2005), it has only one sequon for *N*-glycosylation, which is not located at the glycosyl hydrolase domain of the enzyme. The genes *PADG_07351* and *PADG_07879* translate into sequences of 724 and 593 amino acids, respectively. The calculated pIs were 6.29 and 8.65, with predicted MWs of 77 and 64 kDa, respectively. Prediction of their *N*-glycosylation sites identified two sequons located at the glycosyl hydrolase domain for each *β*-(1,3)-glucanase. The *α*-(1,4)-amylase gene (*PADG_04432*) encoded for a protein of 557 amino acids, with a predicted MW of 61 kDa and a pI of 6.4. Based on primary structure predictions, 2 *N*-glycosylation sites in *α*-(1,4)-amylase were located in glycosyl hydrolase domain. To highlight some features of the studied enzymes, we predicted the 3D structures (Figure 5B) using the I-TASSER server (Roy *et al*., 2010). The PBD template/model accuracies were 2y8kA/–1.15, 1 bhgA/–1.92, 3cnhS/–2.23 and 1qhpA/–0.05 for *PADG_07461*, *PADG_07351*, *PADG_07879* and *PADG_04432*, respectively. A putative glycan structure was inserted into the predicted *N*-glycosylation sites of each protein. The 3D structure model of *α*-(1,3)-glucanase, *α*-(1,4)-amylase and *β*-(1,3)-glucanase (*PADG_07351*) showed that their glycosyl hydrolase domains were predominantly comprised of *α*-helix structures, whereas *β*-strands were found only in the enzymatic domain of the *PADG_07879*-coded *β*-(1,3)-glucanase.

## Discussion

*N*-glycosylation inhibition has previously been shown to impair the growth and morphogenesis of *P. brasiliensis* yeast cells (Dos Reis Almeida *et al*., 2011). Glucans account for approximately 40% of the cell wall of the yeast and mycelial forms (Kanetsuna *et al*., 1969), and in the yeast cell 95% of these glucans are *α*-linked (Kanetsuna *et al*., 1972), whereas in mycelia all glucans are *β*-linked. Moreover, *α*-glucan biosynthesis involves an ordered series of events, which play an important role in the morphology and structural integrity of fungal cells (Dekker *et al*., 2004; Grun *et al*., 2005). Therefore, we investigated whether *N*-glycosylation is involved in *α*- and *β*-(1,3)-glucanase activities, which are critical for cell wall remodelling (Kanetsuna *et al*., 1972; Flores-Carréon *et al*., 1979). We detected high levels of *α*- and *β*-(1,3)-glucanase activities in extracts of yeast cells and mycelia, respectively. These results corroborate with the well-known concept that *α*-glucan is the major glucan in cell wall of *P. brasiliensis* yeasts, whereas the mycelial forms contain only *β*-glucan (Kanetsuna and Carbonell, 1970, Kanetsuna *et al*., 1972; San-Blas *et al*., 1977). We also substantiated that *N*-glycans, although important for the secretion of many proteins, may not be required for *α*- and *β*-(1,3)-glucanase secretion by *P. brasiliensis* yeast and mycelial cells.

Inhibition of *N*-glycosylation induced by addition of TM to fungal cultures also delayed the mycelia-to-yeast and yeast-to-mycelia transitions. This finding corroborates with previous observations that TM treatment impairs fungal morphogenesis and growth, which was evident by the fact that TM-treated cells displayed reduced size and frequency of budding (Dos Reis Almeida *et al*., 2011). We also verified that in the yeast-to-mycelium transition the cells displayed 40% reduced *α*-(1,3)-glucanase activity, although they became endowed with *β*-(1,3)-glucanase activity. Conversely, in the mycelium-to-yeast transition, the cells produced 40% less *β*-(1,3)-glucanase, whereas *α*-(1,3)-glucanase activity was verified. These data suggest that the decrease or increase in glucanase activity depends on the fungal form to which the cells are being transformed.

The deglycosylation of an enzyme most often implies changes in thermal or pH stability as well as modification of kinetic parameters (De Barros *et al*., 2009). As verified for *N*-acetyl-*β*-d-glucosaminidase (NAGase) from yeasts (Dos Reis Almeida *et al*., 2011), *N*-glycosylation inhibition does not modify the optimal pH and temperature for fungal *α*-(1,3)-glucanase, *β*-(1,3)-glucanase or *α*-(1,4)-amylase activities (data not shown). The decreased *α*-(1,4)-amylase activity in the underglycosylated fungal extract suggests that *N*-glycans have a significant influence on enzyme features. As previously verified for NAGase activity, the reduced *α*-(1,4)-amylase activity may account for the impaired growth and morphogenesis in TM-treated *P. brasiliensis* cells, because *α*-(1,4)-amylase from *P. brasiliensis* produces short oligosaccharides acting as primer molecules for the biosynthesis of glucans. These compounds abound in the cell walls of mycelia and yeasts (Kanetsuna *et al*., 1969) and variations in their synthesis or degradation are crucial for the dimorphism of the fungus. Considering the dynamic nature of the metabolism of glucan in the fungal cell wall (Bowman and Free, 2006), further study of the kinetics of glucan formation and degradation in *P. brasiliensis* cells, additional searches for correlation of glucan content and deeper understanding of *P. brasiliensis* morphogenesis are needed. The assumption that underglycosylation is responsible for the lower *α*-amylase activity in samples derived from TM-treated cells was based in our previous observation, performed in similar experimental conditions, on *N*-acetyl-*β*-d-glucosaminidase activity: since we had specific antibodies for the protein detection, we have determined its levels and concluded that the enzyme was similarly secreted in the culture supernatants of TM-treated or untreated yeasts. Then, decreased enzymatic activity of TM-treated yeasts was imputed to underglycosylation itself (Dos Reis Almeida *et al*., 2011). Currently, there is no tool to detect the *α*-amylase levels and we cannot exclude the possibility that decreased synthesis or secretion of *α*-amylase may account for the detection of lower enzymatic activity. Further studies are necessary to validate this inference, or not, concerning *α*-amylase. The prediction of the glycoprotein 3D structure generated low C scores and model accuracy, and the absence of *P. brasiliensis*-related protein structures in the PDB made the reconstruction of high-homology models impossible. The fact that *α*- and *β*-(1,3)-glucanase activities were not critically influenced by TM treatment allows us to infer that the presence of *N*-glycans in single or multiple sites does not interfere crucially with the performance of these enzymes. Indeed, because the *α*-(1,3)-glucanase single sequon for *N*-glycosylation is not located at the enzymatic domain, we can easily explain the absence of effects of TM treatment on enzyme activity. Consistently, the decreased *α*-(1,4)-amylase activity resulting from TM treatment is most likely due to the two sequons found at the glycosyl hydrolase domain of the protein. However, two sequons were also identified in the *β*-(1,3)-glucanases (*PADG_07351* and *PADG_07879*) that were annotated in the genome. Despite this finding, TM treatment did not interfere with enzyme activity. We conclude that the sequons may not be associated with *N*-glycan occupancy. Indeed, whereas the presence of the Asn–X–Ser/Thr sequon is necessary for the receipt of an *N*-glycan, transfer of the *N*-glycan to this sequon does not always occur, owing to conformational or other constraints during glycoprotein folding. Therefore, Asn–X–Ser/Thr sequons are referred to as potential *N*-glycan sites. An estimated two-thirds or more of those sequons are likely to be *N*-glycosylated (Stanley *et al*., 2009). An additional possibility may be considered with the *PADG_07879*-encoded *β*-(1,3)-glucanase. For instance, because its structure is relaxed, deglycosylation is unlikely to induce significant conformational changes and prevent binding to laminarin, which was used as the substrate in our analysis.

Paracoccin, a dual-function lectin/NAGase from *P. brasiliensis* (Dos Reis Almeida *et al*., 2010), plays an important role in fungal growth (Ganiko *et al*., 2007). When underglycosylated by TM treatment, paracoccin, unlike *α*- and *β*-(1,3)-glucanase, showed decreased enzyme activity. Notably, inhibition of *N*-glycosylation had little or no effect on the optimum pH and temperature for both enzymes.

The importance of *N*-glycosylation in enzyme traffic and activity is well reported in the literature. The abolishment of putative *N*-linked glycan sites can reduce protein expression and decrease the catalytic activity of the protein. Underglycosylated enzymes often exhibit only minimal activity compared with their fully glycosylated counterparts. However, deglycosylation of some enzymes reportedly increases their catalytic activity, binding affinity and substrate specificity (Skropeta, 2009). These data highlight not only the singular role of individual *N*-glycans in regulating enzyme function but also the influence of their diversity on enzyme properties (Skropeta, 2009). To date, many aspects of the *N*-linked glycan(s) of this enzyme remain to be assessed. Considering that *N*-glycosylation is crucial for many fungal biological processes and interaction of yeasts with host cells (Dos Reis Almeida *et al*., 2011), and that cell wall polysaccharides may be responsible for key events associated with *P. brasiliensis* virulence (Puccia *et al*., 2011), our findings reveal aspects of *P. brasiliensis* glycobiology that contribute to better comprehension of paracoccidioidomycosis pathogenesis.
